# Population Mobility, Globalization, and Antimicrobial Drug Resistance

**DOI:** 10.3201/eid1511.090419

**Published:** 2009-11

**Authors:** Douglas W. MacPherson, Brian D. Gushulak, William B. Baine, Shukal Bala, Paul O. Gubbins, Paul Holtom, Marisel Segarra-Newnham

**Affiliations:** Migration Health Consultants Inc., Cheltenham, Ontario, Canada (D.W. MacPherson); McMaster University, Hamilton, Ontario, Canada (D.W. MacPherson); Migration Health Consultants Inc., Singapore (B.D. Gushulak); Agency for Healthcare Research and Quality, Rockville, Maryland, USA (W.B. Baine); Food and Drug Administration, Rockville (S. Bala); University of Arkansas for Medical Sciences, Little Rock, Arkansas, USA (P.O. Gubbins); Keck School of Medicine, Los Angeles, California, USA (P. Holtom); Veterans Affairs Medical Center, West Palm Beach, Florida, USA (M. Segarra-Newnham)

**Keywords:** population mobility, migration, antimicrobial resistance, globalization, perspective

## Abstract

Human travel contributes to antimicrobial drug resistance around the world.

Population mobility is a main factor in globalization of public health threats and risks, specifically distribution of antimicrobial drug–resistant organisms. Drug resistance is a major risk in healthcare settings and is emerging as a problem in community-acquired infections. Traditional health policy approaches have focused on diseases of global public health significance such as tuberculosis, yellow fever, and cholera; however, new diseases and resistant organisms challenge existing approaches. Clinical implications and health policy challenges associated with movement of persons across barriers permeable to products, pathogens, and toxins (e.g., geopolitical borders, patient care environments) are complex. Outcomes are complicated by high numbers of persons who move across disparate and diverse settings of disease threat and risk. Existing policies and processes lack design and capacity to prevent or mitigate adverse health outcomes. We propose an approach to global public health risk management that integrates population factors with effective and timely application of policies and processes.

Human mobility is causing an increase in antimicrobial drug–resistant organisms and drug-resistant infectious diseases. International population movement is an integral component of the globalization process. Current population movement dynamics rapidly and effectively link regions of marked health disparity, and these linkages can be associated with risk for importation of drug-resistant infectious diseases.

During the past century, developments in public health sanitation (*1*), infrastructure engineering (*2*), vaccines (*3*), and antimicrobial drugs have contributed substantially to the control of infectious diseases, markedly decreasing associated illness and death. These developments have largely occurred in economically advanced regions and have produced complacency and a belief that the public health threats posed by infectious diseases have been conquered. However, by the early 1990s, infectious diseases were again being identified as substantial domestic and international public health threats in and to western nations (*4*).

Although many infections of clinical relevance are effectively managed with the use of vaccines, antimicrobial drugs, or newer therapies, challenges to the control of infectious diseases remain. These challenges occur in industrialized and in developing countries and result at least in part from the failure of antimicrobial drugs to meet expectations for management and control of disease in clinical and public health contexts. Declining antimicrobial drug effectiveness has current and future consequences that affect all elements of the health sector, e.g., research and development, public health policy, service delivery, and payment programs. The emergence of antimicrobial drug resistance adversely affects patient care and threatens effective management of public health infectious diseases globally (*5*).

Antimicrobial drug failure may occur for many reasons, e.g., reduced adherence to drug therapy, suboptimal dosing, diagnostic and laboratory error, ineffective infection control, counterfeit or altered drugs, and resistance (innate or acquired). Although much attention is focused on resistance patterns of eubacteria (*6*), resistance is being found for virtually all microbial agents including mycobacteria (*7*,*8*), viruses (*9*,*10*), parasites (*11*,*12*), and fungi (*13*,*14*). Antimicrobial drug resistance phenotype is commonly described in terms of the resistance characteristics of the microorganism. These characteristics are either constitutionally based intrinsic characteristics of the organism or resistance factors acquired through induced genetic expression or gene transfer between organisms.

Human activities strongly affect acquired resistance. Emergence of drug resistance in environments that enable sharing of drug-resistance genes between organisms has been documented. Human activities that contribute to ecological niche pressures, such as antimicrobial drug use (*15*) and manufacturing or biological waste disposal into the environment (*16*,*17*), can support the development of resistance.

Against this background of diverse antimicrobial drug resistance, interregional migration and the processes associated with international population mobility can affect the spread and distribution of resistant organisms. These mechanisms of spread become increasingly common when people move among locations with disparate delivery of health services, public health systems, and regulatory frameworks for therapeutic drugs, particularly antimicrobial agents. We describe the role of population mobility in the dispersal of drug-resistant organisms and the emerging need for global standards, programs, and policies in the management of drug resistance, especially for mobile populations.

## Population Mobility and Association with Infectious Diseases and Microbial Resistance

Each year, ≈2 billion persons move across large geographic distances; approximately half cross international boundaries (Table). The International Air Transport Association reported that their members carried 1.6 billion passengers in 2007, among which 699 million flew internationally (*24*). The United Nations World Tourism Organization estimated 924 million international tourist arrivals in 2008 (*19*). International movements for permanent resettlement by immigrants, refugees, asylum seekers, or refugee claimants, and temporary movement by migrant workers and others augment the total international movements each year. The International Labour Organization stated that in 2004, an estimated 175 million persons (3% of the world’s population) lived permanently outside their country of birth and that there were 81 million migrant workers (excluding refugees) globally (*22*).

**Table:** Global estimates of annual migrant populations

Administrative category	Population estimates and year	Reference
Refugees	16 million in 2007	(*18*)
Asylum seekers or refugee claimants	650,000 in 2007	(*18*)
Internally displaced persons	51 million in 2007, includes those displaced by natural disasters and conflict	(*18*)
Temporary (recreational or business travel) movement	924 million in 2008	(*19*)
Regular immigrants	Annual flow of 2.4 million, reported in 2005 (from a stock of 200 million immigrants worldwide)	(*20*)
International students	2.1 million in 2003	(*21*)
Migrant workers	81–86 million in 2005	(*22*)
Trafficked (across international borders) persons	Estimated 800,000 in 2006	(*23*)
Domestic arrivals, by air	Estimated 900 million in 2007	(*24*)

Despite the magnitude of mobile populations, translating international movement statistics into imported disease risk is challenging for several reasons. Domestic surveillance systems generally report disease events and only occasionally refer to infection in the context of place of acquisition. Patients’ travel or migration history may not be routinely gathered as part of the reporting requirements. Nevertheless, considerable information supports the belief that international population mobility plays a role in introducing antimicrobial drug–resistant disease, as follows.

### Human Travel to Disease-Nonendemic or Low Disease–Endemicity Regions

Mobile population importation of drug-resistant infections and diseases is most evident where the expected frequency of the infection or disease is low or absent. For diseases in nonendemic areas, it can be fairly assumed that humans imported the disease. Many examples of imported multidrug-resistant (MDR) infectious diseases are associated with migrant populations, e.g., MDR *Plasmodium falciparum* malaria in immigrants, tourists, and returned foreign-born travelers (*25*–*27*). Tuberculosis in regions of low disease endemicity, such as western Europe and North America, is also related to the influx of persons from tuberculosis-hyperendemic areas (*28*). Tuberculosis in foreign-born persons can shift the local disease epidemiology from endemic to imported and includes the risk for MDR TB (*29*–*32*) and extensively drug-resistant (XDR) TB (*33*,*34*).

### Geographic Tracking of Human-to-Human Transmitted Diseases and Drug Resistance over Time

The emergence of high-level resistance to penicillin G by *Streptococcus pneumoniae*, first described in South Africa in 1977, followed by resistance to multiple drugs is an example of international tracking of human-to-human disease and this organism over almost 4 decades. Modern molecular microbiologic techniques are now being used to confirm its global spread (*35*).

Similar studies have been conducted on the international spread of drug-resistant gonorrhea (*36*,*37*). *Neisseria gonorrhoeae* resistant to penicillin, tetracycline, and multiple other drugs, detected in Southeast Asia during the 1960s and 1970s, has been an emerging public health issue in the United States (*38*,*39*). The reported emergence of quinolone-resistant gonorrhea in the United States (*40*) followed a similar pattern of reactive public health response to the contribution of human mobility to international and then intranational spread. Successive treatment guidelines emphasize the importance of population mobility and the dispersal of resistant organisms in this illness (reference 41 in Technical Appendix). The convergence of a resistant threat with decreased access to effective alternative therapy (cefixime shortage) during 2002–2003 complicated management and control (reference 42 in Technical Appendix). Increasingly, development of clinical management guidelines for diagnosing and treating illness caused by many resistant organisms will refer to international differences in drug-resistance patterns (reference 43 in Technical Appendix).

Since multidrug– or methicillin–resistant *Staphylococcus aureus* (MRSA) was first reported in the United States in 1968, its prevalence in North American healthcare institutions has grown, contributing to increased (number and duration) hospital stays and an associated increased number and severity of cases and more deaths (references 44,45 in Technical Appendix). Recent descriptions of primary community-associated MRSA infections causing death have raised concerns about the control and management of this organism in not only North America but other locales worldwide as well (references 46,47 in Technical Appendix). Clinical and laboratory testing can link distant disease exposures to local isolation of resistant strains (references 48–50 in Technical Appendix). A worrying development of antimicrobial drug resistance in *S. aureus* has been the emergence and geographic extension of reduced susceptibility to vancomycin, which at one time was the reliable backup therapy for MRSA infections (references 51–53 in Technical Appendix). Although MRSA is not uniquely a human pathogen, the nature of its clinical distribution and ability to be carried in asymptomatic persons supports its association with human-to-human transmission over large distances.

### Humans as Asymptomatic Carriers or Mobile Vectors of Antimicrobial Drug–Resistant Organisms

As with MRSA, humans can asymptomatically carry and transmit other cutaneous, enteric, or respiratory microbial flora from zones of high to low prevalence. Some of these organisms may have innate drug resistance or may reflect acquired resistance patterns that are not typical of locally acquired disease. Typhoid disease, *Shigella,* and *Campylobacter* infections are a few of many other enteric infections for which humans are documented carriers (references 54–56 in Technical Appendix).

Recently, the potential for drug-resistant influenza viruses with emergent and pandemic potential has captured considerable global health attention (references 57–59 in Technical Appendix). The local appearance of novel influenza strains with rapid global distribution raises questions about the role of human mobility in the spread and distribution of drug-resistant viruses (reference 60 in Technical Appendix). Although local antiviral drug pressure is associated with rapid appearance of resistance, drug-resistant strains of influenza have also been associated with importation (reference 61 in Technical Appendix).

The role of international tourists, travelers, or migrants colonized with antimicrobial drug–resistant organisms, in terms of transmission potential when they arrive in areas of a low disease prevalence, is difficult to detect and largely unexplored (reference 62 in Technical Appendix). The reality of this risk is illustrated when persons obtain healthcare services outside their normal place of residence. Wounded military personnel and a group often referred to as medical tourists are at increased risk of acquiring nosocomial infections caused by drug-resistant organisms and of subsequently importing their infections when they repatriate to their country of residency.

Additionally, the role of international facilities that provide dental, surgical, medical, diagnostic, and therapeutic services to international travelers is expanding (reference 63 in Technical Appendix). Health services in other countries may be provided in regulatory and standardization environments that differ from those at the patients’ place of origin. The estimated risk for hospital-acquired infections in developing countries is 2–20× greater than that in industrialized countries (reference 64 in Technical Appendix). Antimicrobial drug–resistance patterns may also differ, as may health services, infection control practices, and public health requirements for surveillance and reporting of antimicrobial drug resistance. The extension and transfer of nosocomial infections between regions and within the community has been well documented at the national level (references 65–67 in Technical Appendix). As more high-risk and vulnerable populations travel internationally, either requiring or planning medical or surgical care abroad, or as migrants enter countries seeking healthcare services not available in their own countries, the international consequences of imported drug-resistant infections will be seen more frequently.

In some scenarios, linking the emergence of antimicrobial drug resistance and international mobility can be challenging. Given the global prevalence of many common organisms, their role in causing infections in high-risk populations (e.g., the elderly and patients with concurrent conditions such as diabetes, renal failure, malignancy, or immune compromise or patients who have had abdominal surgery) or certain institutional environments (e.g., intensive care units, burn units, long-term care facilities) may create similar local pressures potentially leading to multifocal emergence of drug resistance. Regardless of whether simultaneous multifocal emergence of resistance is a factor, unaffected areas will be linked to affected areas through mobilization of persons from zones of high to low prevalence. Microbial identification and typing systems, antibiograms, and new technologies for identifying genetic clones and “fingerprints” of microbes are better at defining the origin and patterns of spread of MDR organisms.

Local monitoring of susceptibility patterns combined with knowledge of emerging drug resistance, regionally or internationally, is already recognized as a component of some resistant infections such as MDR TB and XDR TB. Growing population mobility makes local monitoring an increasingly important component of routine surveillance for antimicrobial resistance.

## Role of International Policies, Processes, and Globalization in the Control of Imported Antimicrobial Drug–Resistant Diseases

Since development of the first international maritime sanitation regulations in 1832, coordinated international responses have been required to manage common threats. Such undertakings have always had to balance the benefits of mitigation with the negative effects of disease control interventions on international trade and commerce (reference 68 in Technical Appendix). The modern version of these regulations, the International Health Regulations, focuses on a limited number of diseases and outbreaks of international public health significance for surveillance and reporting but only peripherally addresses population mobility and drug-resistance patterns (reference 69 in Technical Appendix).

The association of international movements of conveyances, goods, and people with introductions of disease and vectors has been long recognized (references 70–71 in Technical Appendix). Human travel, trade, and commerce have frequently been implicated in the redistribution of diseases. Examples include yellow fever in the 18th and 19th centuries, anopheline mosquito malaria vectors in the 1930s, and, more recently, *Aedes albopictus* and dengue, the extension of West Nile Virus infection into North America, and the spread of chikungunya infections in Europe (references 72–76 in Technical Appendix). No specific antimicrobial therapies are available for yellow fever, dengue, West Nile, and chikungunya viruses, among others. Expanding human population mobility will affect and influence the spread, introduction, and endemicity of resistant and untreatable microbes because infections are unequally and rather unpredictably distributed around the world.

## Proposed Approach to Global Public Health Risk Management

As recently demonstrated by influenza A pandemic (H1N1) 2009 virus, the volume, rapidity, and complexity of international movements exceed current international disease control practices (reference 77 in Technical Appendix). Effective responses require engagement of local capacities, standardization of practices, multisectorial partnerships, and rigorous health intelligence with threat and risk assessment. The spread and introduction of resistant infections may not be preventable; but planning, recognition, and coordinated response can mitigate the consequences. Specifically, to control antimicrobial drug resistance and international movement of disease risk associated with human mobility, greater international collaboration and standardization are needed in the following areas:

- Prescriber education, training, and invigilation in terms of antimicrobial drug stewardship for good patient care and reduction of risk for emerging drug resistance.
- Infection control training, certification, and practice.
- Laboratory methods, proficiency testing, and quality management.
- Active and passive surveillance systems, including routine gathering of travel and migration history, rapid analysis, and reporting.
- Engagement of process and regulatory tools unrelated to public health but related to health outcomes, e.g., good manufacturing practices and quality systems for medical devices and pharmaceuticals (references 78,79 in Technical Appendix).
- Pharmaceutical security systems for standard and quality medicines. (The importance of this issue relevant to drug effectiveness, patient safety, and emergence of resistance appeared in a United States Pharmacopeia drug quality report from countries associated with the US Agency for International Development; the report indicated that antibiotic drugs, antimalarial drugs, antituberculous drugs, and antiretroviral agents for treatment of HIV/AIDS were found to be commonly substandard or counterfeit [reference 80 in Technical Appendix]. Even in industrialized countries, counterfeit drugs may enter the marketplace either directly from local illegal producers or through international portals such as importation or Internet pharmacy access [references 81–83 in Technical Appendix.])
- Animal and plant health sector engagement. (Not only do subtherapeutic, subquality antiinfective therapies and low-level environmental antimicrobial drugs affect illness and death at the human level, but they also have the potential for emergence of drug resistance at the microbial level [references 84–88 in Technical Appendix.])

Although all the above-listed efforts are essential, none will be sufficient without integrating the role played by humans and their international movement into modeling the complex relationship with antimicrobial drug resistance and microorganisms (reference 89 in Technical Appendix). Enhanced global surveillance and population mapping demarcating differential zones of disease prevalence and major health disparities will support targeted interventions such as routine drug sensitivity analyses for infections originating in certain situations.

Acknowledging the dynamic role of population mobility in emerging risks to public health is a first step in formulating an effective response, but other components will be needed if this risk is to be successfully mitigated (reference 90 in Technical Appendix). Components of this response will include the following:

- Accurate and robust assessment of threat to risk management based on modern population characteristics that include mobility, travel, and migration history.
- Mitigation of risk through nonhealth partnerships in other sectors, including economics and trade, education, agriculture, and security, all of which will affect the determinants of health, regional disease outcomes, and critical decision making for effective intervention and control.
- Augmenting local knowledge and timely communications related to populations expressing emerging disease threats and risks and linking early detection through diagnostic and confirmatory epidemiologic tools and medical technology.

## Conclusions

Although the association of human movement with antimicrobial drug resistance is not new, the extent of risk to public health caused by population mobility and drug-resistant infections is increasing. A shift in the existing paradigm of pathogen-focused policies and programs would contribute to a healthier future for everyone. The shift should address population mobility as a part of an integrated approach to decrease globalization of infectious disease threats and risks.

## Supplementary Material

Technical Appendix Population Mobility, Globalization, and Antimicrobial Drug Resistance
