# Supplementary material for: Population Mobility, Globalization, and Antimicrobial Drug Resistance
Source: Emerg Infect Dis. 2009 Nov;15(11):1727–31. doi: 10.3201/eid1511.090419 (PMC2857230; doi:10.3201/eid1511.090419)
Supplement: Technical Appendix — Population Mobility, Globalization, and Antimicrobial Drug Resistance [file 09-0419_Techapp-s1.pdf]

Publisher: CDC; Journal: Emerging Infectious Diseases
Article Type: Perspective; Volume: 15; Issue: 11; Year: 2009; Article ID: 09-0419
DOI: DOI: 10.3201/eid1511.090419; TOC Head: Perspective

Population Mobility, Globalization, and Antimicrobial Drug Resistance

Technical Appendix

41. Wang SA, Lee MV, O'Connor N, Iverson CJ, Ohye RG, Whiticar PM, et al. Multidrug-resistant *Neisseria gonorrhoeae* with decreased susceptibility to cefixime—Hawaii, 2001. Clin Infect Dis. 2003;37:849–52. PubMed DOI: 10.1086/377500

42. Workowski KA, Berman SM. Sexually transmitted disease treatment guidelines, 2006. MMWR Recomm Rep. 2006;55(RR11):1–94. PubMed

43. Lynch JP III, Zhanel GG. Escalation of antimicrobial resistance among *Streptococcus pneumoniae*: implications for therapy. Semin Respir Crit Care Med. 2005;26:575–616. PubMed DOI: 10.1055/s-2005-925524

44. Barrett FF, McGhee RF, Finland M. Methicillin-resistant *Staphylococcus aureus* at Boston city hospital. N Engl J Med. 1968;279:448.

45. Klein E, Smith DL, Laxminarayan R. Hospitalizations and deaths caused by methicillin-resistant *Staphylococcus aureus*, United States, 1999–2005. Emerg Infect Dis [cited 2009 Jun 8]. Available from http://www.cdc.gov/EID/content/13/12/1840.htm

46. Gorwitz RJ, Jernigan DB, Powers JH, Jernigan JA; Participants in the Centers for Disease Control and Prevention–Convened Expert's Meeting on Management of MRSA in the Community. Strategies for the clinical management of MRSA in the community: summary of an experts' meeting convened by the Centers for Disease Control and Prevention. March 2006 [cited 2009 Jun 8]. Available from http://www.cdc.gov/ncidod/dhqp/pdf/ar/CAMRSA_ExpMtgStrategies.pdf

47. Friedrich AW, Daniels-Haardt I, Köck R, Verhoeven F, Mellmann A, Harmsen D, et al. EUREGIO MRSA-net Twente/Münsterland—a Dutch-German cross-border network for the prevention and control of infections caused by methicillin-resistant *Staphylococcus aureus*. Euro Surveill. 2008;13(35):pii=18965 [cited 2009 Jun 8]. Available from http://www.eurosurveillance.org/ViewArticle.aspx?ArticleId=18965

48. Bochet M. Francois P, Longtin Y, Gaide O, Renzi G, Harbarth S. Community-acquired methicillin-resistant *Staphylococcus aureus* infections in two scuba divers returning from the Philippines. J Travel Med. 2008;15:378–81. PubMed DOI: 10.1111/j.1708-8305.2008.00243.x

49. Wannet WJ, Heck ME, Pluister GN, Spalburg E, Van Santen MG, Huijsdans XW, et al. Panton-Valentine leukocidin positive MRSA in 2003: the Dutch situation. Euro Surveill. 2004;9(11):pii=484 [cited 2009 Jun 8]. Available from http://www.eurosurveillance.org/ViewArticle.aspx?ArticleId=484

50. Struelens MJ, Hawkey PM, French GL, Witte W, Tacconelli E. Laboratory tools and strategies for methicillin-resistant *Staphylococcus aureus* screening, surveillance and typing: state of the art and unmet needs [review]. Clin Microbiol Infect. 2009;15:112–9. PubMed DOI: 10.1111/j.1469-0691.2009.02698.x

51. Centers for Disease Control and Prevention. Reduced susceptibility of *Staphylococcus aureus* to vancomycin—Japan, 1996. MMWR Morb Mortal Wkly Rep. 1997;46:624–6. PubMed

52. Centers for Disease Control and Prevention. Update: *Staphylococcus aureus* with reduced susceptibility to vancomycin—United States, 1997 MMWR. 1997;46:813–5.

53. Wang G, Hindler JF, Ward KW, Bruckner DA. Increased vancomycin minimal inhibitory concentrations in *Staphylococcus aureus* clinical isolates from a university hospital during a five-year period [Epub 2006 Sep 6]. J Clin Microbiol. 2006;44:3883–6. PubMed DOI: 10.1128/JCM.01388-06

54. Baine WB, Farmer JJ III, Gangarosa EJ, Hermann GT, Thornsberry C, Rice PA. Typhoid fever in the United States associated with the 1972–1973 epidemic in Mexico. J Infect Dis. 1977;135:649–53. PubMed

55. Weissman JB, Marton KI, Lewis JN, Friedmann CT, Gangarosa EJ. Impact in the United States of the Shiga dysentery pandemic of Central America and Mexico: a review of surveillance data through 1972. J Infect Dis. 1974;129:218–23. PubMed

56. Johnson JY, McMullen LM, Hasselback P, Louie M, Jhangri G, Saunders LD. Risk factors for ciprofloxacin resistance in reported *Campylobacter* infections in southern Alberta. Epidemiol Infect. 2008;136:903–12. PubMed DOI: 10.1017/S0950268807009296

57. Baz M, Abed Y, McDonald J, Boivin G. Characterization of multidrug-resistant influenza A/H3N2 viruses shed during 1 year by an immunocompromised child. Clin Infect Dis. 2006;43:1555–61. Epub 2006 Nov 8. PubMed DOI: 10.1086/508777

58. World Health Organization. Avian influenza, including influenza A(H5N1), in humans: WHO interim infection control guideline for health care facilities (revised 10 May 2007, currently under revision) [cited 2009 Jun 8]. Available from http://www.who.int/csr/disease/avian_influenza/guidelines/infectioncontrol1/en

59. Meijer A, Lackenby A, Hungnes O, Lina B, van der Werf S, Schweiger B, et al. Oseltamivir-resistant influenza A (H1N1) virus, Europe, 2007–08 season. Emerg Infect Dis. 2009;15:552–60.

60. World Health Organization. Influenza (H1N1). Update 45 [cited 2009 Jun 8]. Available from http://www.who.int/csr/don/2009_06_08/en/index.html

61. Vicente D, Cilla G, Montes M, Mendiola J, Pérez-Trallero E. Rapid spread of drug-resistant influenza A viruses in the Basque Country, northern Spain, 2000–1 to 2008–9. Euro Surveill. 2009;14(20):pii=19215 [cited 2009 Jun 8]. Available from http://www.eurosurveillance.org/ViewArticle.aspx?ArticleId=19215

62. Leverstein-van Hall MA, Blok HE, Paauw A, Fluit AA, Trolestra A, Mascini EM, et al. Extensive hospital-wide spread of a multidrug-resistant *Enterobacter cloacae* clone, with late detection due to a variable antibiogram and frequent patient transfer.. J Clin Microbiol. 2006;44:518–24. PubMed DOI: 10.1128/JCM.44.2.518-524.2006

63. MacReady N. Developing countries court medical tourists. Lancet. 2007;369:1849–50. PubMed DOI: 10.1016/S0140-6736(07)60833-2

64. Pittet D, Allegranzi B, Storr J, Bagheri Nejad S, Dziekan G, Leotsakos A, et al. Infection control as a major World Health Organization priority for developing countries. J Hosp Infect. 2008;68:285–92. PubMed DOI: 10.1016/j.jhin.2007.12.013

65. Naas T, Coignard B, Carbonne A, Blanckaert K, Bajolet O, Bernet C, et al.; French Nosocomial Infection Early Warning Investigation and Surveillance Network. VEB-1 extended-spectrum beta-lactamase-producing *Acinetobacter baumannii,* France. Emerg Infect Dis. 2006;12:1214–22. PubMed

66. Kassis-Chikhani N, Decré D, Gautier V, Burghoffer B, Saliba F, Mathieu D, et al. First outbreak of multidrug-resistant *Klebsiella pneumoniae* carrying blaVIM-1 and blaSHV-5 in a French university hospital. J Antimicrob Chemother. 2006;57:142–5. PubMed DOI: 10.1093/jac/dki389

67. Turton JF, Kaufmann ME, Gill MJ, Pike R, Scott PT, Fishbain J, et al. Comparison of *Acinetobacter baumannii* isolates from the United Kingdom and the United States that were associated with

repatriated casualties of the Iraq conflict. J Clin Microbiol. 2006;44:2630–4. PubMed DOI: 10.1128/JCM.00547-06

68. Gushulak BD, MacPherson DW. The basic principles of migration health: population mobility and gaps in disease prevalence. Emerg Themes Epidemiol. 2006;3:3. PubMed DOI: 10.1186/1742-7622-3-3

69. World Health Organization. International health regulations 2nd ed. 2005 [cited 2009 Jun 8]. Available from http://whqlibdoc.who.int/publications/2008/9789241580410_eng.pdf

70. Tatem AJ, Hay SI, Rogers DJ. Global traffic and disease vector dispersal. Proc Natl Acad Sci U S A. 2006;103:6242–7. PubMed DOI: 10.1073/pnas.0508391103

71. Tatem AJ. Rogers DJ, Hay SI. Global transport networks and infectious disease spread. Adv Parasitol. 2006;62:293–343. PubMed DOI: 10.1016/S0065-308X(05)62009-X

72. Soper FL, Wilson DB. *Anopheles gambiae* in Brazil: 1930 to 1940. New York: Rockefeller Foundation; 1943.

73. Teixeira MG, Costa MD, Barreto F, Barreto ML. Dengue: twenty-five years since reemergence in Brazil. Cad Saude Publica. 2009;25:S7–18. PubMed

74. Centers for Disease Control and Prevention. West Nile virus update—United States, January 1—August 19, 2008. MMWR Morb Mortal Wkly Rep. 2008;57:899–900. PubMed

75. Straetemans M. on behalf of the ECDC Consultation Group on Vector-Related risk for Chikungunya Virus Transmission in Europe. Vector-related risk mapping of the introduction and establishment of *Aedes albopictus* in Europe. Euro Surveill. 2008;13(7):pii=8040 [cited 2009 Jun 8]. Available from http://www.eurosurveillance.org/ViewArticle.aspx?ArticleId=8040

76. Depoortere E, Coulombier D. ECDC Chikungunya Risk Assessment Group. Chikungunya risk assessment for Europe: recommendations for action. Euro Surveill. 2006;11(19):pii=2956 [cited 2009 Jun 8]. Available from http://www.eurosurveillance.org/ViewArticle.aspx?ArticleId=2956

77. Trifonov V, Khiabanian H, Rabadan R. Geographic dependence, surveillance, and origins of the 2009 influenza A (H1N1) virus. N Engl J Med. 2009;361:115–9. PubMed DOI: 10.1056/NEJMp0904572

78. US Food and Drug Administration, Center for Devices and Radiological Health. Good manufacturing practices (GMP)/quality systems (QS) regulation [cited 2009 Jun 8]. Available from http://www.fda.gov/cdrh/devadvice/32.html

79. World Health Organization. Quality assurance of pharmaceuticals. A compendium of guidelines and related materials. Volume 2. 2nd ed. Good manufacturing practices and inspection 2007 [cited 2009 Jun 8]. Available from http://www.who.int/medicines/areas/quality_safety/quality_assurance/QualityAssurancePharmVol2.pdf

80. Primo-Carpenter J, McGinnis M. Matrix of drug quality reports in USAID-assisted countries. US Pharmacopeia Drug Quality and Information Program [cited 2009 Jun 8]. Available from http://www.usp.org/pdf/EN/dqi/ghcDrugQualityMatrix.pdf

81. US Food and Drug Administration. Combating counterfeit drugs. A report of the Food and Drug Administration. 2004 [cited 2009 Jun 8]. Available from http://counterfeiting.unicri.it/docs/FDA%20combating%20ctf%20drugs.pdf

82. Yankus W. Counterfeit drugs: coming to a pharmacy near you. American Council on Science and Health. 2006 [cited 2009 Jun 8]. Available from http://www.acsh.org/publications/pubID.1384/pub_detail.asp

83. US Food and Drug Administration. FDA news release. FDA finds consumers continue to buy potentially risky drugs over the internet; practice puts consumers at risk and may be more expensive than domestic purchasing. July 2, 2007 [cited 2009 Jun 8]. Available from http://www.fda.gov/NewsEvents/Newsroom/PressAnnouncements/2007/ucm108946.htm

84. Sharma R, Larney FJ, Chen J, Yanke LJ, Morrison M, Topp E, et al. resistance during composting of manure from cattle administered sub-therapeutic antimicrobials. J Environ Qual. 2009;38:567–75. PubMed DOI: 10.2134/jeq2007.0638

85. Pasqualotto AC, Denning DW. Generic substitution of itraconazole resulting in sub-therapeutic levels and resistance. Int J Antimicrob Agents. 2007;30:93–4. PubMed DOI: 10.1016/j.ijantimicag.2006.11.027

86. Delsol AA, Anjum M, Woodward MJ, Sunderland J, Roe JM. The effect of chlortetracycline treatment and its subsequent withdrawal on multi-resistant *Salmonella enterica* serovar Typhimurium DT104 and commensal *Escherichia coli* in the pig. J Appl Microbiol. 2003;95:1226–34. PubMed DOI: 10.1046/j.1365-2672.2003.02088.x

87. Roe MT, Pillai SD. Monitoring and identifying antibiotic resistance mechanisms in bacteria. Poult Sci. 2003;82:622–6. PubMed

88. Lipsitch M, Samore MH. Antimicrobial use and antimicrobial resistance: a population perspective. Emerg Infect Dis. 2002;8:347–54. PubMed DOI: 10.3201/eid0804.010312

89. Arias CA, Murray BE. Antibiotic-resistant bugs in the 21st century—a clinical super-challenge. N Engl J Med. 2009;360:439–43. PubMed DOI: 10.1056/NEJMp0804651

90. Heymann DL. Resistance to anti-infective drugs and the threat to public health. Cell. 2006;124:671–5. PubMed DOI: 10.1016/j.cell.2006.02.009
